# Causes, management and outcomes of polyhydramnios at a secondary level hospital in Cape Town, South Africa

**DOI:** 10.1371/journal.pone.0317256

**Published:** 2025-03-05

**Authors:** Amon Siveregi, Anne Horak, Chantal Stewart

**Affiliations:** 1 Mowbray Maternity Hospital, Cape Town, Western Cape, South Africa; 2 Department of Obstetrics and Gynaecology, University of Cape Town, Groot Schuur Hospital, Cape Town, South Africa; Kasr Alainy Medical School, Cairo University, EGYPT

## Abstract

**Introduction:**

Polyhydramnios is associated with both maternal and fetal adverse outcomes. Idiopathic polyhydramnios, regardless of its severity category, was considered not to be associated with an increase in adverse outcomes. In contrast, when conditions such as congenital and chromosomal abnormalities or diabetes mellitus are detected, neonatal and maternal adverse outcomes can be up to five times higher. We thus aimed to document the outcomes of patients with mild, moderate and severe polyhydramnios according to whether an underlying cause was found and which management protocol was followed.

**Methodology:**

We conducted a retrospective cohort study of all patients with polyhydramnios on ultrasound examination at our secondary hospital between January 1, 2018 and December 31, 2020. Hospital folders were reviewed. We recorded demographic data, information on underlying causes, management and outcomes. We summarised categorical variables using count (percentage). We tested the association between categorical variables using the chi-square test. Statistical significance was set at p < 0.05.

**Results:**

A total of 136 patients with polyhydramnios (80 mild, 42 moderate, and 14 severe) were included. Most cases of polyhydramnios were idiopathic regardless of category [81.2% (65/80), 78% (32/42) and 78% (11/14) in the mild, moderate and severe groups, respectively]. The likelihood of occurrence of the composite adverse outcome, was higher with increasing severity of polyhydramnios, with 6.75%. 19.05%, and 35.71% in the mild, moderate and severe groups, respectively, having the composite adverse outcome (p =  0.01). Elective delivery before 40 weeks’ gestation for polyhydramnios in patients with idiopathic polyhydramnios was associated with a significant reduction in the occurrence of the composite adverse outcome compared to awaiting spontaneous labor (3.77% versus 15.79%, p =  0.036),.

**Conclusions:**

Adverse outcomes were related to severity of the polyhydramnios and were significantly lower in the mild compared to the moderate and severe groups, with the rate of adverse outcome in the mild group comparable to that of the general population. Early delivery before 40 weeks gestation may be associated with benefit in moderate and severe groups of polyhydramnios.

## Introduction

### Background

Polyhydramnios, defined as excessive accumulation of amniotic fluid, has an estimated incidence of 0.2%–3.9% [1]. It is associated with both maternal and fetal adverse outcomes. Dye dilutional techniques are the most accurate predictor of amniotic ﬂuid volume.Using this method, a small volume of sodium aminohippurate is injected into the amniotic fluid via ultrasound-guided amniocentesis. The dye is allowed to distribute throughout the amniotic fluid before a small sample is withdrawn to measure the concentration of sodium aminohippurate. By comparing the concentrations of sodium aminohippurate before and after the injection, the volume of the amniotic fluid can be calculated [2]. However, their invasive nature limits their clinical use. Semi-quantitative ultrasonography methods like the amniotic fluid index and single deepest pool are often used [1,3].

Polyhydramnios can be classified into mild (25–29.9 cm), moderate (30–34.9 cm) and severe (>35 cm) groups based on the amniotic fluid index at ultrasonography [4]. The volume of amniotic fluid at any stage of gestation depends on the balance between its production from fetal urine, lung and oral secretions and its uptake through fetal swallowing and intermembranous absorption [5].

### Causes

Approximately 50%–60% of cases of polyhydramnios are unexplained and referred to as idiopathic [1,3]. One systematic review estimated that an underlying cause is found in only 17% of cases in mild polyhydramnios, compared to 91% in moderate to severe polyhydramnios [1]. The commonly listed causes of polyhydramnios include fetal malformations, maternal diabetes mellitus, multiple pregnancies, fetal anaemia, fetal gastrointestinal malformations, neuromuscular conditions and viral and bacterial infections [1]. Approximately 18.8% of pregnant women with diabetes mellitus have polyhydramnios [1]. Other rare causes, such as placental tumours and Bartter syndrome have also been documented in the literature [6].

Laboratory investigations used to identify the aetiology include glycosylated haemoglobin (HbA1c) and oral glucose tolerance test to exclude diabetes mellitus; screening for maternal toxoplasmosis, cytomegalovirus, and parvovirus; and screening for atypical antibodies and the Betke-Kleihauer test to check for feto-maternal haemorrhage [3]. Furthermore, a detailed fetal anatomy scan should be performed to check for structural abnormalities. According to a review done by Karkhanis and Patni in 2014, chromosomal abnormalities are known to be present in 10% of fetuses with sonographic anomalies and polyhydramnios, but in only 1% when ultrasound is considered normal [3]. In cases where fetal malformation or soft markers are present, fetal karyotyping with microarray tests and gene sequencing may be advised to detect chromosomal abnormalities and other rarer conditions, such as Bartter syndrome [1]. In low- and middle-income countries patients often present late for antenatal care and receive their first ultrasound scan beyond the late second trimester. This reduces the ability to investigate them further, hence lower incidences of underlying causes of the polyhydramnios may be reported in these circumstances [7]. Karyotyping may also not be offered at advanced gestation, depending on the termination policies of the country.

### Complications

Maternal dyspnoea, preterm labour, premature rupture of membranes, abnormal fetal presentation, placental abruption, postpartum haemorrhage and umbilical cord prolapse have been described as possible obstetric complications due to uterine overdistention [8–11]. Polyhydramnios has also been shown to be associated with higher birth weights, higher rates of caesarean sections for fetal indications, low neonatal 5-minute APGAR scores, higher rates of admission to neonatal intensive care units (NICU) and increased perinatal mortality [12,13].

### Interventions

Some obstetricians routinely admit patients with polyhydramnios for observation and some will electively deliver them before 40 weeks’ gestation [14,15]. They argue that, if labour and rupture of membranes occur in areas where prompt actions can be taken to avoid any potential complications, maternal and fetal outcomes may be better. However, no studies have shown any benefits to these practices when there is no obstetric indication [3].

### Aims

We thus aimed to assess maternal and perinatal outcomes in patients with polyhydramnios. Specifically, we compared the causes and perinatal outcomes between mild and moderate to severe polyhydramnios. Additionally, we evaluated the perinatal outcomes of patients who underwent induction of labor before 40 weeks’ gestation solely for idiopathic polyhydramnios against those who waited for spontaneous labor.

## Materials and methods

We conducted a retrospective cohort study comparing causes, management and outcomes in women with mild, moderate, and severe polyhydramnios diagnosed on ultrasound scan after 24 gestational weeks from January 1, 2018 to December 31, 2020. Women with an AFI >  25, who followed up and delivered at Mowbray Maternity Hospital (MMH) or Groot Schuur Hospital (GSH) and had complete information in their files were included. Files with incomplete information and those of women who delivered in hospitals outside of our drainage area were excluded.

At our facility, all pregnant patients undergo testing for ABO and Rh antibodies, as well as syphilis and HIV at the first visit. While clinician practices may vary, patients diagnosed with polyhydramnios via ultrasound are typically scheduled for a 75g oral glucose tolerance test and a TORCH screen if there are clinical or ultrasound signs of infection. Those with severe polyhydramnios, particularly when accompanied by fetal anomalies or maternal discomfort, are referred to our tertiary hospital for further evaluation. At the tertiary hospital karyotyping would be offered for patients with fetal anomalies with a high association with chromosomal anomalies.

Some clinicians choose to admit all patients with polyhydramnios for inpatient management starting at 36 weeks, continuing until induced delivery between 38 and 40 weeks gestation. This approach aims to mitigate risks associated with cord prolapse or placental abruption during labor.

Our sonographers are experienced in conducting detailed anatomy scans and consistently identify patients with abnormalities during ultrasound examinations. They use the four-quadrant method to measure the Amniotic Fluid Index (AFI). For any patient with an AFI greater than 25, efforts are made to check for signs of fetal infection, as well as placental and fetal anomalies. These patients are flagged for the attending doctors, and the measurement is recorded in the ultrasound register.

### Sampling technique

A total of 163 ultrasound reports with AFI > 25 were identified in the study period. Of these, 60 reported moderate and severe polyhydramnios and the remainder reported mild polyhydramnios. After applying the inclusion and exclusion criteria, 80 with mild polyhydramnios, 42 with moderate, and 14 with severe polyhydramnios were included. Most of the files that were excluded were either missing or had incomplete information. A total of 136 patient folders were analysed.

### Measures

Demographic data, causes of polyhydramnios, management strategies, and adverse maternal and fetal outcomes were collected from maternal and neonatal case records. The fasting 75g oral glucose tolerance test was utilized to screen for gestational diabetes, while pre-existing diabetes was identified through self-reporting or diagnosed based on random blood sugar levels and elevated HbA1c before 16 weeks of gestation. Polyhydramnios was classified as idiopathic if all available tests returned negative results. A composite adverse outcome was defined as the occurrence of any adverse event, whether maternal or fetal. Maternal adverse events included premature rupture of membranes, preterm labour, placenta abruptio, cord prolapse among others, while neonatal adverse events included low APGAR scores, admission to neonatal ICU, hypoxic ischaemic encephalopathy e.t.c.

Details of the data are presented in S1 Fig .

### Statistical considerations

#### Sample size.

Sample size was calculated using estimates of event rates for composite outcomes from previous studies [16–19]. The event rate in the mild polyhydramnios group was estimated to be 10%, while in the moderate to severe group, it was estimated to be 28%. Based on these estimations we calculated sample sizes of 72 per group (total of 144) to achieve an 80% power to detect a difference of 18% of the composite outcome at an alpha level of 0.05. These computations were done based on the chi-square test of the null hypothesis that there was no difference between the moderate +  severe and mild groups. The sample size was calculated using STATA 15 software

#### Statistical analysis.

We summarised continuous variables using the mean (standard deviation) and categorical variables using count (percent). We reported the proportion (percentage) with the composite outcome with the corresponding 95% confidence intervals. We tested the association between categorical variables using the chi-square test. Statistical significance was set at p < 0.05. STATA 15 was used. We compared the incidence of composite adverse outcomes between the three severity categories of polyhydramnios. Additionally, the composite adverse outcome was compared between different management strategies, specifically between patients electively delivered before 40 weeks’ gestation for polyhydramnios and those who delivered spontaneously or due to other obstetric reasons.

#### Ethical considerations.

Approval for the study was granted by the University of Cape Town’s Human Research Ethics Committee, approval number (HREC REF 213/2021). Permission to collect data was obtained from the relevant authorities at MMH. Individual patient consent was waived as this was a retrospective study. Hospital numbers were the only patient identifiers on data extraction sheets.

## Results

We included 136 patients in the study. Demographic data are shown in Table 1. There was no difference in the demographic characteristics between the 3 groups.

**Table 1:** Demographic Data of the Cohort (n =  136).

	Mild polyhydramnios	Moderate polyhydramnios	Severe polyhydramnios	p-value
Age mean(range)	29.6 (17–44)	30.3 (18–42)	28.9 (22–42)	0.25
BMI mean (SD)	30.1 (7.1)	32.1 (8.5)	33 (6.3)	0.523
Diabetes mellitus				
(Preexisting/gestational) n (%)	12 (15)			0.963
HPT n (%)	7 (15.2)	2 (13.4)	1 (20.0)	0.600
Parity median (range)	2 (0–6)	2 (0–3)	2 (0–8)	0.235
HIV positive n (%)	15 (19)	11 (26)	1 (14)	0.519
Atypical antibodies n (%)	4 (5)	0	0	0.411
Gestational age at diagnosis median (IQR)	33.9 (27–43)	34.5 (24 – 400	32.8 (27–39)	0.641

AFI, amniotic fluid index; HIV, human immunodeficiency virus; HPT, hypertension.

### Causes.

Most cases of polyhydramnios were idiopathic regardless of category (81.2% in the mild group and 77.2% in the moderate + severe group). The commonest associated cause of polyhydramnios was diabetes (pre-existing or gestational), occurring in 15% (12/80) in the mild polyhydramnios group, 16.7% (7/42) in the moderate group, and 14.3% (2/14) in the severe group. There was no statistical difference between the groups (p =  0.963).

The likelihood of detecting a fetal anomaly increased with the increasing AFI category, with 2.86% (2/70) anomalies being detected in the mild polyhydramnios group, 9.1% (3/33) in the moderate polyhydramnios group, and 21.4% (3/14) in the severe group (p = 0.03). See Table 2.

**Table 2:** Fetal Congenital Abnormalities.

Fetal Anomaly	Gestational Age at Diagnosis (weeks)	Polyhydramnios Group
6 fingers bilaterally	28	Mild
Dilated renal pelvis bilaterally	33	Mild
Dilated renal pelvis bilaterally	32	Moderate
Talipes	36	Moderate
Short long bones	29	Moderate
Atrioventricular septal defects micrognathia, hypotelorism	38	Severe
Hydrops fetalis	28	Severe
Micrognathia, rocker bottom feet	27	Severe

No patients underwent fetal karyotyping. Most of the detected anomalies were not strongly associated with chromosomal abnormalities. The few cases with anomalies known to be linked to chromosomal issues declined karyotyping for cultural or religious reasons and presented late in the third trimester. Only two patients had TORCH screen done and both of them were negative.

### Management protocols.

Only two patients (both from the mild polyhydramnios group) had cervical lengths measured to predict preterm labour and both were normal (>25mm). For the patients that had follow-up scans, the chance of AFI improving on subsequent scans was inversely related to the AFI category with 22.5% cases of mild polyhydramnios improving, 16,7% of moderate polyhydramnios, and 14% of severe polyhydramnios cases improving (p =  0.05). The likelihood of being admitted to the ward for observation from 36 weeks of gestation increased with increasing AFI category, with 10% (8/80) women in the mild polyhydramnios group being admitted, 19% (8/42) in the moderate group, and 35.7% in the severe group being admitted (p =  0.03). There was no significant difference in delivery before 40 weeks gestation by AFI category, with 39.4% (30/80) women in the mild group of polyhydramnios being delivered before 40weeks, 59.5% (25/42) in the moderate group, and 50% (7/14) of patients in the severe group being delivered before 40 weeks gestation(Table 3).

**Table 3:** Management Protocol Comparisons between the 3 Groups (n =  136).

	Mild polyhydramnios	Moderate polyhydramnios	Severe polyhydramnios	p-value
Follow up scan n (%)	54 (67.5)	16 (38)	9 (64)	0.07
Polyhydramnios improvement	18 (22.5)	7 (16.7)	2 (14)	0.05
Admitted to ward from 36w	8 (10)	8 (19)	5 (35.7)	0.03
Delivered between 38 – 40 wk	30 (39.4)	25 (59.5)	7 (50)	0.09
OGTT done	69 (86)	37 (88)	12 (85)	0.911

### Outcomes

The likelihood of occurrence of the composite adverse outcome, incidence of preterm labour or preterm premature rupture of the membranes (PPROM), admission to NICU and need for assisted ventilation were all significantly higher with increasing AFI category (Table 4). There were no cases of abruptio placentae recorded. Only one case of cord prolapse was recorded (in the mild group). There was no significant difference in the occurrence of 5 min APGAR scores < 7, hypoxic ischaemic encephalopathy (HIE) and shoulder dystocia between the 3 groups (Table 4).

**Table 4:** Comparison of Outcomes between Mild and Moderate ±  Severe Groups of.

	Mild polyhydramnios	Moderate polyhydramnios	Severe polyhydramnios	p-value
Preterm labour	3 (3.75)	5 (11.9)	4 (28)	0.007
PPROM	1 (1.25)	4 (9.52)	2 (14.2)	0.03
Cord prolapse	1 (1.25)	0	0	0.70
Placenta abruption	0	0	0	–
PPH	1 (1.25)	1 (2.38)	0	0.79
Shoulder dystocia	1 (1.25)	2 (4.76)	0	0.382
5min Apgar < 7	2 (2.5)	2 (4.76)	1 (7.14)	0.60
Admission to NICU	2 (2.5)	4 (9.51)	4 (28.57)	0.002
Need for assisted ventilation	2 (2.5)	4 (9.52)	3 (21.43)	0.02
HIE	0	1 (2.38)	2 (7.14)	0.07
Birth trauma	1 (1.25)	0	0	1.00
Composite adverse outcome	6 (7.5)	8 (19.05)	5 (35.71)	0.01

PPROM, preterm premature rupture of the membranes; NICU, neonatal intensive care unit; HIE, hypoxic ischaemic encephalopathy.

### Idiopathic polyhydramnios and early delivery before 40 weeks gestation

Delivery before 40 weeks for polyhydramnios in patients with idiopathic polyhydramnios was associated with a significant reduction in the occurrence of the composite adverse outcome compared to awaiting spontaneous labor (3.77% [2/51] versus 15.79% [9/48], p =  0.036), respectively. Delivery before 40 weeks was not associated with reduced adverse composite outcome rate in the mild polyhydramnios group, while there is a statistically significant reduction in adverse outcomes for moderate idiopathic polyhydramnios patients delivered before 40 weeks. Delivery before 40 weeks for severe idiopathic polyhydramnios also shows benefit, but the p-value was not significant as there were zero adverse outcomes in the other group. The subgroup analysis by AFI category is shown in Table 5 below.

**Table 5:** Idiopathic polyhydramnios and early delivery, by AFI category.

	Delivery before 40weeks	Spontaneous labour	P – value
Mild polyhydramnios n (%)	1 (3.70)	2 (5%)	0.80
Moderate polyhydramnios n (%)	1(5.0)	5 (41.0)	0.01
Severe polyhydramnios n (%)	0 (0.0)	2 (40.0)	0.08

## Discussion

Our study showed that the majority of cases of polyhydramnios in women attending low risk antenatal clinic were idiopathic, regardless of the severity of the polyhydramnios. Furthermore, the study demonstrated that the increase in adverse outcomes above the background rate for general pregnant mothers is associated with the severity of polyhydramnios rather than whether it is idiopathic or not.

Maternal diabetes was the commonest cause associated with polyhydramnios, occurring in 15.0%, 16.6%, and 14.2% of the mild, moderate, and severe polyhydramnios groups, respectively. This is comparable to results from most published studies and justifies performing an OGTT in all cases of polyhydramnios [1,20].

Separate studies by Backley et al, Dashe et al, Hamza et al and Hendricks et al, all showed the prevalence of congenital anomalies in severe polyhydramnios to be in excess of 75% [1,15,21,22]. The anomaly rate in the mild group of polyhydramnios ranged between 17%–29%. Our study, however, showed that the anomaly rate in the moderate and severe group was only 9.1% and 21.4% respectively, and that in the mild group was 2.8%, with most of them being minor and possibly incidental. The anomaly rates in the mild polyhydramnios group were comparable to that in the general population of pregnant mothers. Despite our study confirming that anomaly rates increase with severity of polyhydramnios, the rates were significantly lower compared to those from studies mentioned earlier. As patients in low- middle-income countries such as ours often only have their first ultrasound scan late in pregnancy, all severity categories of polyhydramnios were typically diagnosed after the median gestational age of 33 weeks in our study. This could limit pick-up rates of fetal anomalies. It is also important to consider that some of the studies we reviewed were conducted in tertiary institutions where patients were referred due to underlying maternal conditions or concerning features on ultrasonography.

We only performed two TORCH screens, and both came back negative. A study by Fayyaz et al showed that TORCH screen may not be necessary, especially in patients with isolated polyhydramnios, with no ultrasound features suggestive of infection [23]. In our institution it has also been reserved for such cases where ultrasound features suggestive of congenital infection are present. No cases of alloimmunisation were recorded in our study.

There is general consensus that pregnancies complicated by polyhydramnios are at increased risk of adverse outcomes. However, there is conflicting evidence on whether idiopathic polyhydramnios increases rates of these adverse outcomes. Most studies in the literature that have looked at adverse outcomes in patients with idiopathic polyhydramnios do not categorise it into severity groups. Our study has shown that mild polyhydramnios has adverse outcomes no worse than those of the general population of low risk mothers without polyhydramnios [6/80 (7.5%)] [24]; however, complications in the moderate and severe group were up to four times higher [8/42 (19.04%), 5/14 (35.7%)] respectively.

Three studies showed no increase in the risk of adverse outcomes apart from increased caesarean section rates in patients with idiopathic polyhydramnios compared to the general population [10,19,25]. However, these studies did not stratify idiopathic polyhydramnios into severity classes. Conversely, two other studies showed an increase in both caesarean section and postpartum haemorrhage rates [26,27]. These studies also did not stratify polyhydramnios into severity categories. Increased rates of intrauterine fetal deaths (IUFD) at every gestation were reported in patients with idiopathic polyhydramnios compared to those without [25]. Similarly, another study reported an increased risk of IUFD in women with mild polyhydramnios [28]. Wiegand et al. categorised idiopathic polyhydramnios into severity groups. In their study, all severity groups were associated with increased caesarean section rates, low five-minute APGAR scores in the neonates and higher rates of admission to NICU [29]. They also showed an increased rate of adverse outcomes with increasing severity of the polyhydramnios. This differed from our findings in that women with mild polyhydramnios did not have an increase in adverse outcomes compared to other low-risk mothers without polyhydramnios. Furthermore, the study demonstrated that the increase in adverse outcomes above the background rate for general pregnant mothers is associated with the severity of polyhydramnios rather than whether a cause is identified.

There is conflicting evidence regarding antenatal surveillance of pregnancies complicated by polyhydramnios. The American College of Obstetrician and Gynaecologists recommends no increase in surveillance for mild idiopathic polyhydramnios [30]. However in patients with moderate and severe polyhydramnios, they recommend weekly antenatal fetal surveillance starting at 32 to 34 weeks of gestation [30]. Our findings would agree with this approach, as adverse outcomes in the mild polyhydramnios group did not increase beyond the baseline of the general population, while these outcomes were up to five times higher in the moderate and severe groups. Pasquini et al also found no increase in adverse outcome rates in patients with mild idiopathic polyhydramnios, compared to general population of pregnant mothers, and recommended no increase in antenatal fetal survellaince in this group, except when they are in labor [27]. Another study by Matĕcha et al also showed increased adverse outcomes only in the moderate and severe groups of polyhydramnios, recommending increased antenatal surveillance only for these categories [31]. Conversely, a study by Pagan et al showed increased risk of intrauterine fetal demise in mild idiopathic polyhydramnios, hence recommending increased fetal surveillance in all categories of polyhydramnios. However, there have been no randomised control trials to guide how surveillance should be done.

The timing of delivery in these pregnancies is also contentious. Backley et al. showed an increase in caesarean section rates in women managed expectantly compared to those delivered at 38 weeks, but other adverse outcomes were not increased. Our study revealed that there was no benefit in early delivery before 40 weeks compared to waiting for spontaneous labor in patients with mild idiopathic polyhydramnios. However, early delivery did reduce the occurrence of composite adverse outcomes in patients with moderate and severe categories of polyhydramnios. The society of Maternal and Fetal Medicine in America recommends awaiting spontaneous delivery in mild polyhydramnios, while induction is recommended between 37and 38 weeks in the moderate and severe groups to reduce adverse outcomes [30]. Conversely, a study by Pagan et al has shown increased adverse outcomes even in patients with mild polyhydramnios [28]. However, no randomised control trials have been done to determine the optimal timing for patients with different categories of polyhydramnios.

In our study, the most significant complication of polyhydramnios was preterm labour. Studies in this regard are inconclusive, with some finding no increase in preterm birth (PTB), while others reported a 20–50% increase in PTB in pregnancies with polyhydramnios compared to those without [10,29]. These studies did not stratify the polyhydramnios into severity categories. Our study found that PTB was five times higher in pregnancies complicated by moderate +  severe polyhydramnios compared to those with mild polyhydramnios. The PTB rate in the mild group was comparable to the rate in other low-risk pregnancies. Although Karahanoglu et al. also reported a five times increase in PTB rate, this was for all women with idiopathic polyhydramnios [32]. Many et al. reported that the underlying cause of polyhydramnios rather than the relative excess of amniotic fluid determined the occurrence of preterm labour [9]. Our findings suggest, however, that the severity of polyhydramnios is associated with preterm labour regardless of whether or not a cause was found.

There was one case of cord prolapse and no cases of placental abruption in our study. A meta-analysis by Khazaei et al. that included 10 studies, showed that pregnancies complicated by polyhydramnios have an increased risk of placental abruption compared to low-risk pregnancies, with an odds ratio close to 2 [33]. Many clinicians admit patients to await delivery at 38 weeks’ gestation to prevent these complications, but our results suggest that this may not be justifiable due to economic and social costs associated with hospital admission. Our study may have been underpowered to detect differences in the occurrence of placental abruption between the different severity categories of polyhydramnios. We did not find any comparative studies quoting the incidence of cord prolapse in polyhydramnios.

## Study limitations

Due to limited resources in our facility in a low- and middle-income country (LMIC), we lacked access to certain genetic tests for patients with polyhydramnios. At our tertiary facility, these tests are reserved for patients with ultrasound anomalies highly associated with chromosomal abnormalities. This may have led to an overestimation of the proportion diagnosed with idiopathic polyhydramnios. Our sample size was based on an estimated prevalence of mild polyhydramnios at 10%, but the actual prevalence was found to be 6.75%. Additionally, the small number of severe polyhydramnios patients in the study may have limited our ability to detect differences in outcomes between the groups. While the amniotic fluid index (AFI) is a subjective measure, we believe it did not significantly impact our results, as the same sonographers performed scans across all severity categories.

## Conclusion

Most cases of polyhydramnios in our secondary level hospital were idiopathic, regardless of category. Adverse outcomes were related to severity, rather than whether the cause of polyhydramnios is identified, and are significantly lower in the mild group compared to the moderate and severe groups. The rate of adverse outcome in the mild group is comparable to the background risk in the general population. Early delivery between 38 and 40 weeks gestation may be indicated in cases of moderate and severe polyhydramnios polyhydramnios, although larger studies may be needed to confirm this.

## Supporting information

S1 Fig Data collection sheet. (DOCX)

S2 Fig Excel spread sheet. (XLSX)
