# Supplementary material for: Causes, management and outcomes of polyhydramnios at a secondary level hospital in Cape Town, South Africa
Source: PLoS One. 2025 Mar 5;20(3):e0317256. doi: 10.1371/journal.pone.0317256 (PMC11882055; doi:10.1371/journal.pone.0317256)
Supplement: S1 Fig — (DOCX) [file pone.0317256.s001.docx]

**Data Collection Sheet**

**Demographics**

| **Measure** | **Result** |
| --- | --- |
| Age |  |
| Parity |  |
| HIV status |  |
| Atypical antibody status |  |
| Syphilis serology |  |
| AFI |  |
| Gestational age at first detection of polyhydramnios |  |

**Causes**

| **Cause** | **Yes** | **No** | **Not done** |
| --- | --- | --- | --- |
| Gestational/overt diabetes mellitus |  |  |  |
| Fetal congenital anomalies |  |  |  |
| Fetal chromosomal abnormalities |  |  |  |
| Toxoplasmosis |  |  |  |
| Parvovirus |  |  |  |
| Rubella |  |  |  |
| Cytomegalovirus |  |  |  |
| Other causes |  |  |  |

**Management**

| **Management** | **Yes** | **No** |
| --- | --- | --- |
| Cervical length surveillance |  |  |
| Follow-up scan |  |  |
| Did polyhydramnios improve |  |  |
| Did polyhydramnios worsen |  |  |
| Admitted to ward |  |  |
| Induction of labour between 38 and 40weeks |  |  |
| Amniodrainage |  |  |
| Indomethacin |  |  |
| Sulindac |  |  |
| OGTT or random blood sugar |  |  |

OGTT, oral glucose tolerance test

| Cervical length measurement | Lengths and gestation measurements were made. | Number of times cervical length measured | Interval between cervical length measurement |
| --- | --- | --- | --- |
| Yes/No |  |  |  |

**8.1.4 Maternal Outcomes**

| **Outcome** | **yes** | **No** |
| --- | --- | --- |
| Preterm labour/GA at occurrence |  |  |
| PPROM/PROM/ GA at occurrence |  |  |
| Malpresentation |  |  |
| Abruptio placenta/GA at occurrence |  |  |
| Post-partum haemorrhage |  |  |
| Shoulder Dystocia |  |  |
| Composite outcome |  |  |
| Cord prolapse |  |  |

GA, gestational age; PPROM, preterm prelabour rupture of membranes; PPROM, prelabour rupture of membranes

**8.1.5 Fetal Outcomes**

| Outcome | Yes | No |
| --- | --- | --- |
| 5 minute APGAR < 7 |  |  |
| Admission to NICU |  |  |
| Need for Assisted ventilation |  |  |
| Any Sign of Hypoxic Ischaemic Encephalopathy |  |  |
| Birth Trauma |  |  |
| Birth Weight |  |  |
| Composite Outcome |  |  |

NICU, neonatal intensive care unit
